# Metastasis of ovarian cancer to nasal skin and skin on the trunk: a rare case report

**DOI:** 10.3389/fonc.2023.1266820

**Published:** 2023-10-18

**Authors:** Chen Chen, Ouyang Yingyao, Xiang Yan, He Qianru, Wang Hong, Chen Chen, Yang Lei

**Affiliations:** ^1^ Department of Cancer Center, The First Hospital of Jilin University, Changchun, Jilin, China; ^2^ The Medical Department, Jiangsu Simcere Diagnostics Co., Ltd, Nanjing, China; ^3^ Nanjing Simcere Medical Laboratory Science Co., Ltd, Nanjing, China; ^4^ The State Key Laboratory of Translational Medicine and Innovative Drug Development, Jiangsu Simcere Diagnostics Co., Ltd, Nanjing, China

**Keywords:** cutaneous metastases, neoplasm metastasis, ovarian cancer, skin metastases, treatment

## Abstract

Cutaneous metastases of ovarian cancer are rare and often have poor prognosis. We report a case of a 62-year-old woman with recurrent low-grade serous ovarian cancer, who presented with lung, brain, and multiple skin (nasal and anterior chest wall) metastases approximately six months after the initial diagnosis. In this case, Nijmegen breakage syndrome carrier status caused by RAD50 heterozygous mutation and previous bevacizumab therapy could be the predisposing factor for cutaneous metastases. The patient was treated with local radiotherapy (nasal skin and brain, 30Gy/6f/1.2W) and three courses of chemotherapy with albumin-bound paclitaxel and carboplatin, resulting in drastic remission of the cutaneous metastases. Unfortunately, treatment interruption resulted in rapid tumor progression, followed by death. This case represents an interesting example of cutaneous metastasis of ovarian cancer with rare clinical manifestations, unique genetic mutations, and reasonable response to treatment. Chemoradiotherapy might be an appropriate option for cutaneous metastases of ovarian cancer. Nevertheless, we still hope to find out the best treatment strategy after collecting and reviewing more cases in the future.

## Background

Ovarian cancer is the most lethal gynecologic malignancy owing to the often late diagnosis and associated widespread metastases at the initial diagnosis itself (1). Ovarian cancer generally metastasizes throughout the peritoneal cavity, including the omentum, intra-abdominal pelvic, and para-aortic lymphatic metastasis. Additionally, in some patients, ovarian cancer metastasizes to the pleura, lungs, liver, and lymph nodes via lymphatic channels and the hematogenous route (2). Cutaneous metastases from visceral malignancies are rare; the same is true for ovarian cancer as well. A study in the Netherlands has shown that breast carcinoma (50%), lung carcinoma (13.6%), and gastrointestinal tumors (7.3%) are the most common primary tumors resulting in cutaneous metastases in women (3). Cutaneous metastasis of ovarian cancer is rare, representing only 3.3% of all causes (4). It is most often located on the abdomen or thorax (5), and typically predicts a poor prognosis for the patient.

Here, we present an unusual case of metastatic ovarian cancer associated with nasal cutaneous manifestations. We intended to further elucidate the diagnosis and therapy for these rare but unfortunate cases.

## Case presentation

A 61-year-old gravida 1 para 1 postmenopausal Chinese woman presented to the clinic with a distending pain in her lower abdomen since past six months. Her medical history and family history was not significant. She had a history of type 2 diabetes, cholecystitis, and glaucoma.

Physical examination revealed massive greater ascites. Serum carbohydrate antigen 125 (CA125) was greater than 1000 U/mL, and abdominal ultrasound revealed pelvic masses. The patient had undergone bilateral salpingo-oophorectomy, omentectomy, pelvic lymph node dissection, and appendectomy in July 2021. Postoperative pathology indicated right ovarian low-grade serous cystadenocarcinoma that measured 10cm × 9cm × 5 cm, and pelvic lymph nodes tested negative for cancer. Therefore, the final diagnosis was right ovarian low-grade serous cystadenocarcinoma, FIGO (International Federation of Obstetrics and Gynecology) stage IA. Consequently, she was treated with three cycles of adjuvant chemotherapy with liposome-paclitaxel and carboplatin. One month after the end of chemotherapy, in November 2021, the patient experienced a relapse with multiple metastases in both lungs, which was confirmed by a percutaneous biopsy. Serum CA125 and HE4 levels were within the normal range. Treatment with liposomal doxorubicin and cyclophosphamide seemed to be effective after two courses, and no BRCA (Breast Cancer) pathogenic mutation was detected in the peripheral blood. She was then administered one cycle of liposomal doxorubicin + gemcitabine + bevacizumab followed by two cycles of cisplatin + gemcitabine + bevacizumab, but unfortunately, her treatment and reexamination were interrupted due to the COVID-19 pandemic. The last chemotherapy she had was in March 2022.

**Figure 1:**
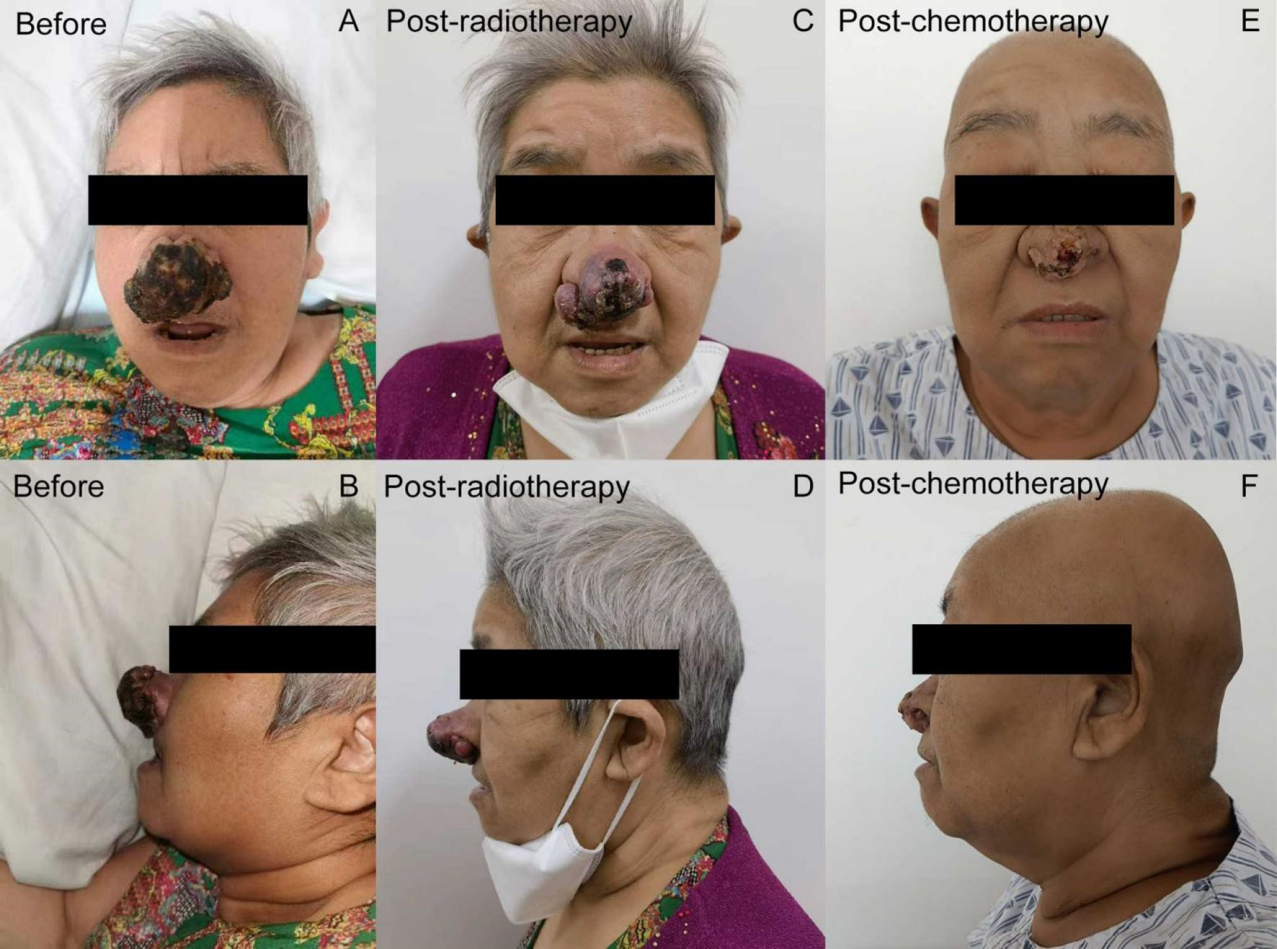
Treatment-related nasal lesion changes of the patient **(A, B)** A palpable, exophytic, rough lesion with central ulceration and serous crust located on the tip of the nose (at primary admission). **(C, D)** After radiation therapy with the dose of 30 Gy, the long diameter of the nasal tip tumor decreased to 5 cm, with partial pain relief. **(E, F)** The long diameter of tumor decreased from 5 to 1.5 cm after the first course of chemotherapy.

Two months later, in May 2022, she presented with a solitary, erythematous, palpable, painless nodule, 0.5-cm in size, located on the nasal tip. Within two months, the painless nodule rapidly evolved into a cauliflower-like, painful, 6-cm size mass. On examination we noticed a palpable, exophytic, rough lesion with central ulceration and serous crust located on the tip of her nose (**Figures 1A, D**). Simultaneously, multiple palpable skin masses were detected all over her body, including the trunk and limbs. Additionally, multiple brain metastases were visible on brain magnetic resonance imaging. Levels of serum CA125 and HE4 remained normal throughout the course of the disease. Biopsies of the nasal and chest skin exhibited a poorly differentiated morphology, which made it difficult to ascertain whether the metastatic lesions had indeed originated from ovarian cancer. To confirm the diagnosis and seek precision therapy, further novel histopathological examinations and genetic tests on both the initial and recurrent tumors were performed. Immunohistochemical (IHC) staining of the skin biopsy sample was negative for p40, PAX8, WT1, ER, and PR, and were positive for Ki-67(+70%), p16, p63, Villin, CK20, CK7, and P53(+60%). IHC analysis of pulmonary metastases revealed the following results: CA125(−), p16(+), CK7(+), WT-1(−), CR(−), PR(−), ER(−), CK5/6(+), Ki-67(+70%), p40(−), TTF1(−), and NapsinA(−). Primary resection ovarian cancer specimens were negatively stained with PAX2, PTEN, Brg-1, WT-1 and p40, and positively stained with p53, ER(+5%), PR(+<25%), p16, and CK5. These results demonstrated that the primary origin of the skin metastases would be ovarian cancer.

Whole exome sequencing (WES) based on next-generation sequencing (NGS) of the peripheral blood samples identified RAD50 heterozygous mutation, suggesting that the patient was a carrier of Nijmegen breakage syndrome. The genomic mutation profiling of nasal formalin-fixed and paraffin-embedded specimens is summarized in **Table 1**. Molecular landscapes are suggestive of a preference for metastasis from ovarian cancer (6, 7), consistent with the pathology results. PIK3CA p.H1047R was detected (30.92% abundance), indicating an opportunity for targeted therapies. Meanwhile, the tumor was microsatellite-stable, and the tumor mutation burden was 2.65 mutations/Mb, which indicated that the patient would not benefit from immunotherapy.

**Table 1:** Genomic mutation profiling of this patient.

Gene name	Mutationcopy	Abundance/ copy
*PIK3CA*	exon21 c.3140A>G p.H1047R	30.92%
*PTEN*	Copy number variation	0.65
*ARID1A*	exon1 p.G87* c.259G>T	82.61%
*ADGRA2*	exon7 p.R263Q c.788G>A	46.89%
*BCOR*	exon10 p.N1459S c.4376A>G	45.80%
*PRDM1*	intron6. c.1902 + 5G>A	38.46%
*PHF6*	exon8 p.V268Tfs*5 c.802_803del	37.54%
*CTNNB1*	exon3 p.D32Y c.94G>T	36.15%
*NSD1*	exon19 p.R1984* c.5950C>T	35.45%
*HIST1H1C*	exon1 p.A197_K201del c.590_604del	34.72%

Due to the size of the lesion, the patient’s general condition, and multiple distant metastases observed in her lungs, skin, and brain, the patient was deemed unfit for surgery, and was treated with local radiotherapy (nasal skin and brain, 30 Gy/6 f/1.2 W) and chemotherapy with albumin-bound paclitaxel and carboplatin. After radiation therapy at the dose of 30 Gy, the long diameter of the nasal tip tumor decreased to 5 cm and offered partial pain relief (**Figures 1B, E**). Following one course of chemotherapy, the patient sustained reductions in the size of her nasal and chest wall lesions (**Figures 1C, F**). Hence, she continued treatment with two more courses of chemotherapy, as they seemed beneficial for her. Unfortunately, chemotherapy had to be interrupted since she suffered from gallstone disease with biliary duct calculi, which required Endoscopic Retrograde Cholangiopancreatography (ERCP). Postoperatively, the patient was in very poor general condition, with lapses in consciousness, and she was sent to a nursing home for palliative care. Following cessation of chemotherapy, the volume of the bilateral lung metastasis lesion increased rapidly and multiple *de novo* metastatic lesions appeared on the anterior chest wall, indicating progressive disease. The patient died three months after the interruption of chemotherapy due to multiple organ failure.

## Discussion

Cutaneous metastasis of ovarian cancer is rare. Given the ovary’s anatomical location, majority of cutaneous metastases are observed around the pelvic region, especially along surgical scars (2). In recent years, some rare skin metastatic sites such as the shoulder (8), scalp (8), neck (9), vulvovaginal area (8), and limbs (10, 11) have also been reported. There is currently only one reported case of ovarian cancer presented with nasal cutaneous metastasis as the initial and main clinical manifestation, reported by Antonio et al. in 2016 (12). Our patient was unusual since the initial presentation was of relatively early, stage IA ovarian serous cystadenocarcinoma, which rapidly developed into an extensive distant disease and rare skin metastases that were remote from the surgical incision. Ovarian cancer can metastasize to the skin through several pathways: contiguous spread, direct implantation, lymphatic spread, extraocular extension, and hematogenous spread (5). Hematogenous and lymphatic pathways are almost certainly the principal associated avenues of spread of ovarian cancer to distant sites. Thus, the mechanism by which ovarian cancer metastasizes to the nasal skin is poorly defined. Considering that the patient developed pulmonary metastasis before the nasal cutaneous manifestations appeared, we strongly conjecture that ovarian cancer cells can be transported through the pulmonary vessels and the lymphatic system to distant locations.

An important factor of skin metastasis in ovarian cancer, to be considered, is tumor histology. High-grade serous carcinomas and endometrioid carcinomas often develop intraperitoneal metastasis. Clear cell carcinomas develop chemotherapy-refractory skin metastases, even in patients with early-stage diseases. Aggressive histotypes, such as undifferentiated carcinomas and neuroendocrine carcinomas, usually develop distant metastases through hematogenous dissemination. Low-grade serous carcinomas also develop skin metastases, which generally occur at locations of surgical scars (13). Furthermore, low-grade serous carcinomas are characterized by a younger age of onset, relative chemoresistance, and better prognoses compared to high-grade serous carcinomas (14). However, in our case, the patient with low-grade serous cystadenocarcinoma experienced a rapid and severe disease progression after first lung metastasis, and she also responded well to chemotherapy. It may be because transplanted tumor cells acquired the malignant transformation during metastasis. It also imposes enormous difficulties on the pathomorphological diagnosis of ovarian cancer skin metastasis.

Comprehensive genomic profiling has been performed in several previous studies to gain a better understanding of the molecular features of metastases. In our case, gene alterations in *PIK3CA*, *PTEN*, *ARID1A*, and *RAD50* indicate that the metastasis might be from the ovary based on the molecular landscape from Catalogue of Somatic Mutations in Cancer data, on the basis of tissue type and histology (https://cancer.sanger.ac.uk/cosmic/), which was proven as metastasis of ovarian cancer histologically.

Since the patient suffered a rapid distant recurrence and disease progression after stopping chemotherapy, we suspect that the metastasis was associated with specific cancer gene mutations. RAD50 heterozygous mutation was detected from the results of WES, suggesting that the patient was the carrier of Nijmegen breakage syndrome (NBS). RAD50, a component of Mre11-Rad50-Nbs1(MRN), plays significant roles in the detection and signaling of DNA double-strand breaks, as well as the repair pathways of homologous recombination and non-homologous end joining (NHEJ) (15), which is significantly associated with enhanced ovarian cancer risk (16). Nevertheless, the impact of PARPi response of germline RAD50 mutation is still unknown. Multiple studies have demonstrated that, although NBS is an autosomal recessive disorder, heterozygous carriers are associated with a higher risk of cancer and are also radiosensitive (17–19), which perhaps explains the rapid tumor recurrence and significant positive radiotherapy effect for our patient. It also has important implications in the treatment decisions, as external beam radiotherapy might be a feasible and efficient treatment option for extensive skin metastases and localized chemotherapy-resistant lesions with minimal morbidity (20, 21). PIK3CA mutaion provides an opportunity for targeted therapies in the PI3K/AKT/mTOR pathway (22, 23).

There are no standard treatment protocols for ovarian cancer associated with spread or isolated skin metastases. In the case of focal cutaneous disease, surgical resection should be a priority (24). However, in most cases, cutaneous metastasis usually develops in the late stages of disease progression, when multiple metastases occur. This renders surgical excision impractical and non-beneficial. Furthermore, unfortunately, skin metastases develop generally after the administration of a chemotherapeutic regimen (25), which renders them relatively resistant to any further cytotoxic drugs. Our patient suffered recurrence after one month of the last platinum-containing regimen, and responded poorly to multiple treatment regimens, including chemotherapy drugs and bevacizumab in follow-up treatment. All of the above reasons resulted in limited treatment options for the patient. Recent data suggests that immune checkpoint inhibitors, immunostimulatory agents such as Imiquimod, and chemotherapy regimens with taxane and bevacizumab may be potentially effective therapeutic approaches (5, 8, 9, 25). However, a different point of view is that anti-VEGF (vascular endothelial growth factor) antibodies, such as bevacizumab, could increase the incidence of brain and skin metastases by eliciting tumor adaptation and progression (26–28). Robinson, W.R et al. has reported that patients with ovarian cancer treated with bevacizumab as secondary therapy after intraperitoneal/intravenous chemotherapy as initial treatment had an increased incidence of extraperitoneal metastases, including cutaneous tissue metastasis (29). Therefore, in the case of our patient, we could not completely exclude the possibility that previous bevacizumab therapy might also have played a role in the development of her cutaneous metastases. Our patient ultimately decided to receive chemotherapy with albumin-bound paclitaxel and carboplatin for financial reasons. Although the patient was classified as platinum-resistant, platinum-based chemotherapy was still administered, because as per the latest evidence, patients with a treatment-free interval for platinum-based chemotherapy (TFIp) <6 months still have a reasonable chance to respond to further platinum-based chemotherapy (30). Fortunately, the metastasis sites in our patient showed a significant positive response to chemotherapy. It is also a reminder that gene heterogeneity between different metastatic sites of ovarian cancer could merit future concerns.

As a stage IV disease, patients with ovarian cancer who develop skin metastases have a universally poor prognosis. It has been reported that the overall survival time after diagnosis of skin metastasis from epithelial ovarian cancer was four months (2–65 months) (9). Generally speaking, the longer the interval between the first surgery and skin metastasis, the longer is the survival time for the patient (8). Our patient experienced disease progression in the skin ten months following the surgical resection, and yet lived for another seven months after receiving additional antitumor therapies, with an overall survival of 17 months. Rapid disease progression caused by treatment interruptions is the underlying cause of death. Early recognition of a cutaneous metastasis and adherence to individualized antitumor therapy might help slow down disease progression, thereby prolonging survival.

This article presents a rare case of ovarian cancer with nasal skin metastasis, underscoring the significance of NGS technology in precision treatment (31). While similar case has been reported previously, the complexity of this patient’s condition highlights the critical role of NGS technology. NGS not only allows for in-depth exploration of genetic variations in tumors, enabling personalized treatment plans and improved treatment outcomes, but also holds profound potential in early cancer diagnosis and targeted drug development. This case emphasizes the broad prospects of NGS technology in future cancer therapy, offering the promise of better treatment options for patients and driving advancements in cancer research and treatment.

## Conclusions

In conclusion, this is a rare case report of an ovarian cancer patient presenting with nasal skin metastasis. NBS carrier status caused by RAD50 heterozygous mutation and the previous bevacizumab therapy might have promoted the occurrence of cutaneous metastases for the patient. Unfortunately, such cutaneous metastases are frequently inoperable and herald a poor prognosis. Our patient exhibited an excellent response to chemoradiotherapy, which provides a promising option for ovarian cancer patients with nasal skin metastasis. Furthermore, NGS analysis suggests the origin of the metastasis, and PI3K inhibitors might serve as a treatment in further clinical treatment.

## Data availability statement

The raw data supporting the conclusions of this article will be made available by the authors, without undue reservation.

## Ethics statement

The studies involving humans were approved by Ethics Committee of the First Hospital of Jilin University. The studies were conducted in accordance with the local legislation and institutional requirements. The participants provided their written informed consent to participate in this study. Written informed consent was obtained from the individual(s) for the publication of any potentially identifiable images or data included in this article.

## Author contributions

CC (1st author): Conceptualization, Formal Analysis, Project administration, Writing – original draft. OY: Writing – original draft. XY: Data curation. HQ: Resources. WH: Methodology, Visualization. CC (6th author): Investigation, Visualization. YL: Supervision, Writing – review & editing.
